# Integrated probability of coronary heart disease subject to the −308 tumor necrosis factor-alpha SNP: a Bayesian meta-analysis

**DOI:** 10.7717/peerj.1236

**Published:** 2015-09-15

**Authors:** C. Sofia Carvalho

**Affiliations:** Institute of Astrophysics and Space Sciences, University of Lisbon, Lisbon, Portugal; Research Center for Astronomy and Applied Mathematics, Academy of Athens, Athens, Greece

**Keywords:** Bayesian meta-analysis, Coronary heart disease, −308 TNF-alpha

## Abstract

We present a meta-analysis of independent studies on the potential implication in the occurrence of coronary heart disease (CHD) of the single-nucleotide polymorphism (SNP) at the −308 position of the tumor necrosis factor alpha (TNF-alpha) gene. We use Bayesian analysis to integrate independent data sets and to infer statistically robust measurements of correlation. Bayesian hypothesis testing indicates that there is no preference for the hypothesis that the −308 TNF-alpha SNP is related to the occurrence of CHD, in the Caucasian or in the Asian population, over the null hypothesis. As a measure of correlation, we use the probability of occurrence of CHD conditional on the presence of the SNP, derived as the posterior probability of the Bayesian meta-analysis. The conditional probability indicates that CHD is not more likely to occur when the SNP is present, which suggests that the −308 TNF-alpha SNP is not implicated in the occurrence of CHD.

## Introduction

Coronary heart disease (CHD) is now widely accepted to consist of a chronic inflammatory disease (Hansson, 2005). CHD is a complex disease with multifold etiology, with both genetic and environmental factors contributing to its occurrence and development.

Among the genetic factors potentially implicated in the emergence of CHD, the tumor necrosis factor alpha (TNF-*α*) has attracted a great interest for its involvement in the inflammatory response of the immune system (Vassali, 1992). There is evidence that TNF-*α* is implicated in an increased susceptibility to the pathogenesis of a variety of diseases. In particular, high serum levels of TNF-*α* affect endothelial cell hemostatic function and hence may modify the risk for developing CHD (Plutzky, 2001). There is also the suggestion that the TNF-*α* gene affects the modulation of lipid metabolism, obesity susceptibility and insulin resistance, thus being potentially implicated in the development of CHD (see Vourvouhaki & Dedoussis, 2008 and references therein).

Among the several single-nucleotide polymorphisms (SNPs) that have been identified in the human TNF-*α*, the best documented one is at the position −308 of the TNF-*α* gene promoter. This SNP involves the substitution of guanine (G) for adenine (A) and the subsequent creation of two alleles (TNF1(A) and TNF2(G)) and three genotypes (GG, GA and AA) (Wilson et al., 1992). It has been hypothesised that the TNF-*α* SNP could change the susceptibility to CHD. However, the results on its association with CHD are contradictory, some implying different influence of the two alleles on the prevalence of CHD, others implying no association (see Zhang et al., 2011 and references therein).

In order to infer the risk of CHD derived from potential risk factors, it is important to develop a formalism that infers correlations among different intervening factors and combines independent data sets for a consistent inference of the correlations. In Vourvouhaki & Carvalho (2011) we introduced a formalism based on Bayesian inference to infer the correlation of the occurrence of CHD with two risk factors and tested a simplistic model for the signal pathway on the three-variable data set from Vendrell et al. (2003). In this manuscript, we extend the formalism to extract information from the combination of data from independent studies and to quantify the combined risk of occurrence of CHD from the −308 TNF-*α* SNP.

The most exhaustive meta-analysis to date on this correlation is the frequentist analysis in Zhang et al. (2011) covering Caucasian, Asian, Indian and African populations. This meta-analysis found a 1.5 fold increased risk of developing CHD when the SNP is present in the Caucasian population, but found no association in the other ethnicities. A more recent meta-analysis, covering the same data sets, found no association in the Caucasian or in the Asian population (Chu et al., 2013).

**Figure 1:**
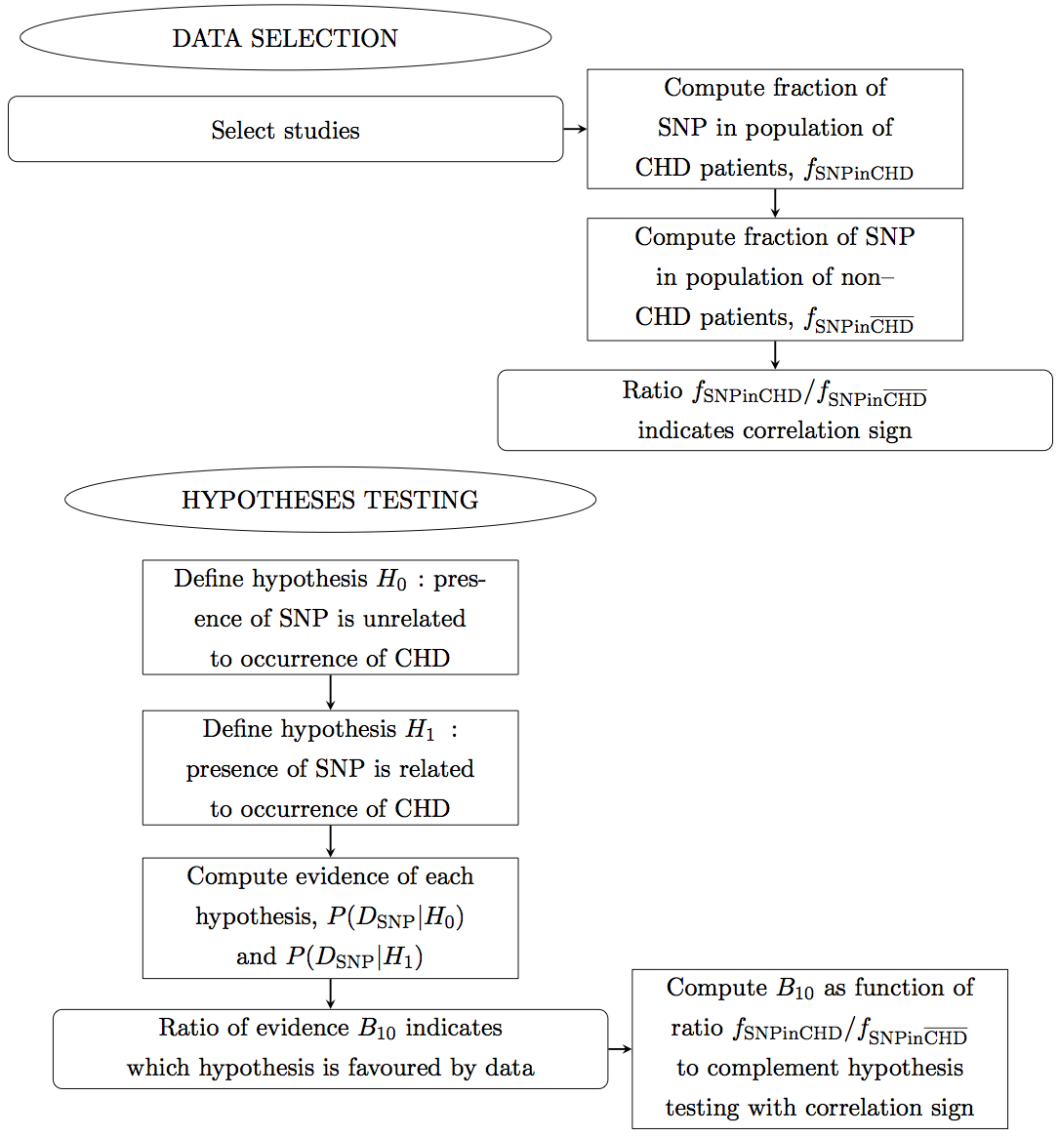
Flow chart. Panel 1 of 3. Ellipses indicate the main actions. Rectangles indicate detailed actions. Rectangles with rounded corners indicate the main results.

**Figure 2:**
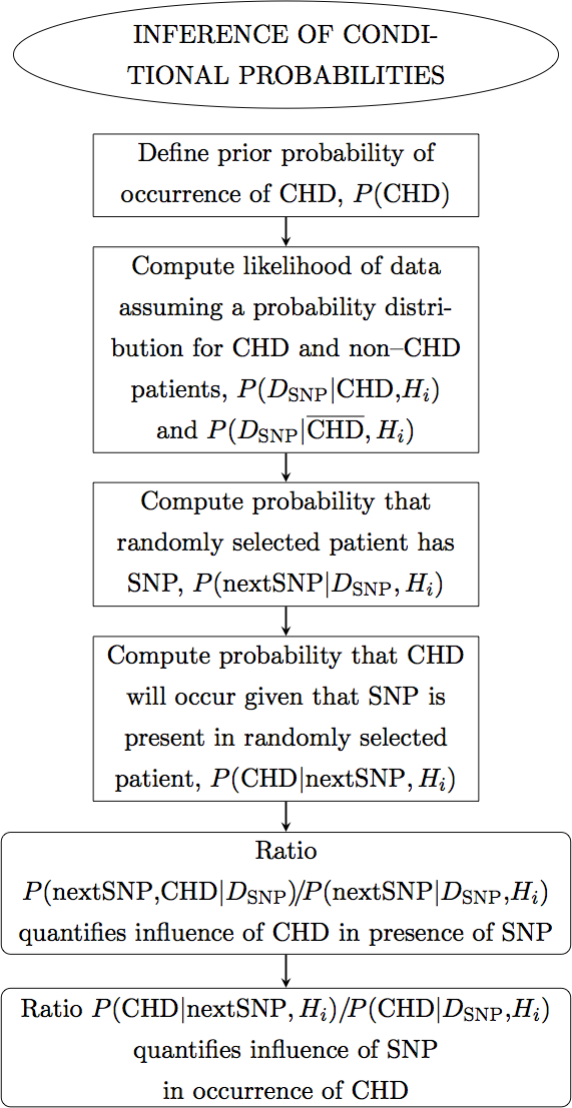
Flow chart. Panel 2 of 3.

**Figure 3:**
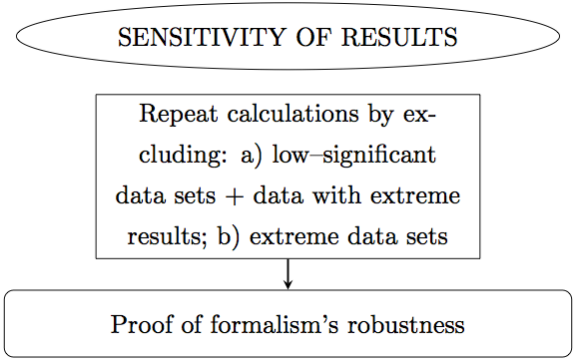
Flow chart. Panel 3 of 3.

In this manuscript we propose a meta-analysis based on Bayesian analysis in an attempt to establish the potential implication of −308 TNF-*α* SNP in the occurrence of CHD. This manuscript is organized as follows. In ‘Methods’ we describe the method. In particular, in ‘Data selection’ we describe the data sets selected; in ‘Hypotheses testing’ we propose two hypotheses and test which best and most simply describes the data. In ‘Results’ we perform the Bayesian analysis of the selected data sets, combined by ethnicity and CHD phenotype, and present the results. In particular, in ‘Inference of conditional probabilities’ we infer the conditional probabilities for the occurrence of CHD given the presence of the SNP; in ‘Sensitivity of the results’ we test the sensitivity of this formalism to low-significance data sets, to data sets with extreme results and to extreme data sets. Finally in ‘Conclusions’ we draw the conclusions. Below there follows a flow chart describing summarily the reasoning of this meta-analysis (Figs. 1, 2 and 3).

## Methods

### Data selection

This analysis is based on twenty data sets (indexed *i*) on two CHD phenotypes (indexed *j*) selected from the studies compiled in Zhang et al. (2011), following a well-documented study identification, data acquisition and selection strategy, including also statistical tests (Hardy–Weinberg equilibrium, heterogeneity, publication bias). The selected data sets are the studies that report the genotypes of both CHD patients and non-CHD (control) patients for the two CHD phenotypes separately. In particular, there were included: fifteen data sets from studies on Caucasians, where six studies are on the CHD phenotype coronary stenosis (CS) (Allen et al., 2001; Elahi et al., 2008; Georges et al., 2003; Sbarsi et al., 2007; Szalai et al., 2002; Vendrell et al., 2003) and nine studies are on the CHD phenotype myocardial infarction (MI) (Antonicelli et al., 2005; Bennet et al., 2006; Dedoussis et al., 2005; Herrmann et al., 1998; Koch et al., 2001; Padovani et al., 2000; Tobin et al., 2004; Tulyakova et al., 2004); and five data sets from studies on Asians on the CHD phenotype coronary stenosis (Hou et al., 2009; Liu et al., 2009; Shun et al., 2009; Li et al., 2003; Chen et al., 2001). The rejected data sets are: three studies on Caucasians (for not reporting data on non-CHD patients); four studies on Asians (three for not reporting data on non-CHD patients and one for not separating the CHD phenotypes); the study on Indians and the study on Africans (both for not separating the CHD phenotypes).

**Table 1:** Data sets and results of hypothesis testing. Column 1: Studies selected for the meta-analysis. The index (A) indicates that a possible association was measured in the original publication; the index (NA) indicates that no association was measured in the original publication. Column 2: The phenotype of the patients in the studies grouped by ethnicity. Columns 3–6: Genotypic frequencies of TNF*α*-308 in CHD patients and non-CHD (control) patients from twenty studies (indexed *i*) and for two CHD phenotypes (indexed *j*), namely coronary stenosis (CS) and myocardial infarction (MI). Columns 7–8: The Bayes factors for the hypotheses considered, for each data set $({H}_{1}^{i,j}/{H}_{0}^{i,j})$, and for the meta-data set of each CHD phenotype $({H}_{1}^{j}/{H}_{0}^{j})$.

Study	Phenotype	CHD patients		Controls		Bayes factor	
(*i*)	(*j*)	GG	GA/AA	GG	GA/AA	$({H}_{1}^{i,j}/{H}_{0}^{i,j})$	$({H}_{1}^{j}/{H}_{0}^{j})$
Allen et al. (NA)	Cauc CS	127	53	222	107	0.14 ± 0.05	
Elahi et al. (A)		59	38	41	54	3.54 ± 1.12	0.049 ± 0.014
Georges et al. (A)		613	236	222	92	0.08 ± 0.03	0.041 ± 0.016^*^
Sbarsi et al. (A)		175	73	185	56	0.33 ± 0.11	
Szalai et al. (A)		229	89	181	87	0.19 ± 0.07	0.048 ± 0.019^**^
Vendrell et al. (A)		231	110	159	48	1.33 ± 0.46	
Antonicelli et al. (A)	Cauc MI	224	69	246	64	0.12 ± 0.04	
Bennet et al. (A)		799	368	1,037	460	0.05 ± 0.02	
Dedoussis et al. (A)		206	31	227	10	26.14 ± 8.56	0.026 ± 0.011
Herrmann et al.^a^ (NA)		325	120	376	158	0.11 ± 0.04	
Herrmann et al.^b^ (NA)		117	79	97	79	0.19 ± 0.06	0.035 ± 0.015^*^
Koch et al. (NA)		565	228	244	96	0.07 ± 0.03	0.030 ± 0.012^**^
Padovani et al. (A)		120	28	114	34	0.17 ± 0.06	
Tobin et al. (A)		365	182	337	168	0.07 ± 0.03	
Tulyakova et al. (NA)		242	64	177	69	0.60 ± 0.21	
Chen et al. (NA)	Asian CS	29	11	21	9	0.27 ± 0.08	0.151 ± 0.057
Hou et al. (NA)		268	32	802	103	0.05 ± 0.02	
Li et al. (NA)		66	8	138	20	0.12 ± 0.04	0.114 ± 0.043^*^
Li et al. (A)		234	52	142	34	0.10 ± 0.03	0.103 ± 0.037^**^
Shun et al. (A)		54	19	118	20	1.10 ± 0.34	

**Notes.**

a French cohort.

b Irish cohort.

* Excluding Elahi et al. (2008), Dedoussis et al. (2005) and Chen et al. (2001), respectively for each phenotype.

** Excluding Georges et al. (2003), Bennet et al. (2006) and Hou et al. (2009), respectively for each phenotype.

The data consist of frequencies of occurrence of the −308 TNF-*α* SNP in randomly selected CHD patients and non-CHD patients, respectively *n*_SNP,CHD_ and ${n}_{\text{SNP},\overline{\text{CHD}}}$. The data are summarized in Table 1 (columns 3–6). The errors indicated were computed from error propagation. Assuming that the methods for measuring the presence of the SNP have a success rate of *r*_suc_ = 0.88 (Sharifian, 2010), and furthermore that the error of a counting result is given by the Poisson approximation $\sqrt{n}$, then the error of a counting result *n* on the presence of the SNP is given by $(1-{r}_{\mathrm{suc}})\sqrt{n}/2.$

**Figure 4:**
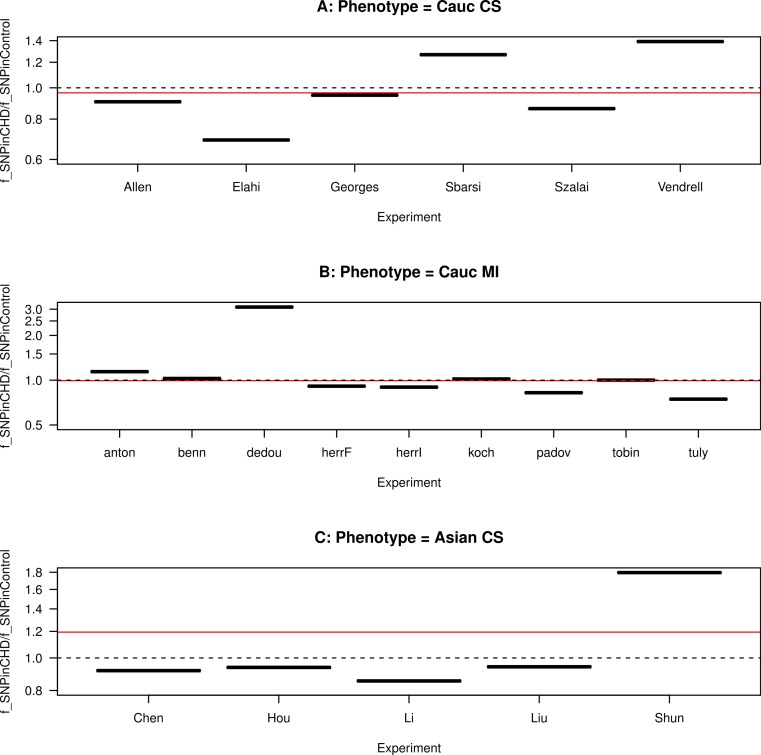
Funnel plot for the ratio of SNP fractions. The ratio of the fraction of SNP in the population of CHD patients to the fraction of SNP in the population of non-CHD patients, ${f}_{\text{SNPinCHD}}/{f}_{\text{SNPin}\overline{\text{CHD}}}$, for each study, grouped by ethnicity and CHD phenotype. (A) Caucasians with coronary stenosis; (B) Caucasians with myocardial infarction; (C) Asians with coronary stenosis. The solid horizontal line is the ratio of the combined data sets included in each panel. The dashed horizontal line marks the ratio equal to one.

**Figure 5:**
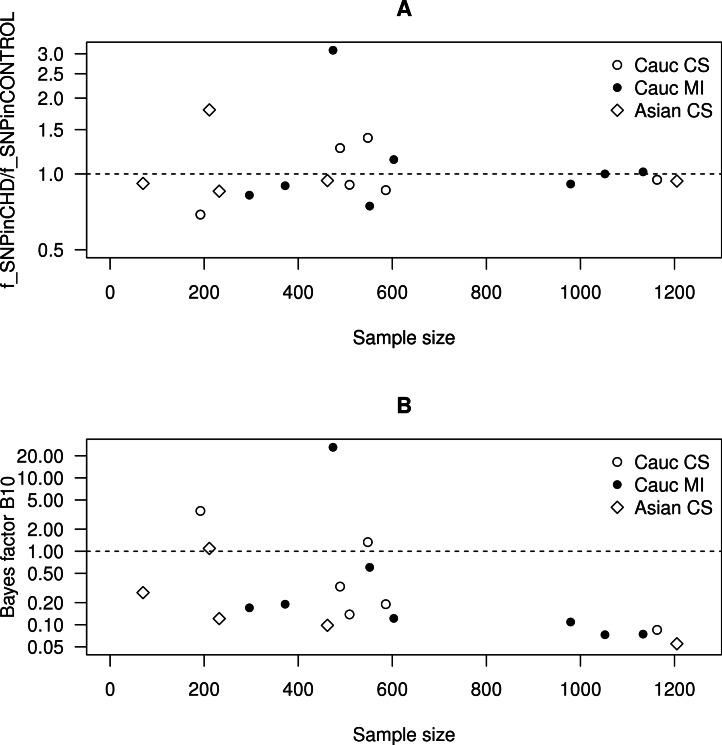
Scatter plots as a function of the sample size. (A) The ratio of the frequency of SNP in the CHD population to the frequency of SNP in the non-CHD population as a function of the sample size. (B) The Bayes factor for the two hypotheses discussed in the text as a function of the sample size.

#### Data heterogeneity

In order to investigate the heterogeneity in the data sets, we compare the size of the effect (defined as a measure of the difference between CHD and non-CHD patients) in each study (Walker, Hernandez & Kattan, 2008). As a measure of the size of the effect, we use the fraction of SNP in the population of CHD patients and in the population of non-CHD patients, respectively *f*_SNPinCHD_ = *n*_SNP,CHD_/*n*_CHD_ and ${f}_{\text{SNPin}\overline{\text{CHD}}}={n}_{\text{SNP},\overline{\text{CHD}}}/{n}_{\overline{\text{CHD}}}$, where ${n}_{\text{CHD}}={n}_{\text{SNP},\text{CHD}}+{n}_{\overline{\text{SNP}},\mathrm{CHD}}$ is the total number of CHD patients and ${n}_{\overline{\text{CHD}}}={n}_{\mathrm{SNP},\overline{\text{CHD}}}+{n}_{\overline{\text{SNP}},\overline{\text{CHD}}}$ is the total number of non-CHD patients. Moreover, the ratio of these two fractions gives an indication of the correlation sign. Hence, if ${f}_{\text{SNPinCHD}}/{f}_{\text{SNPin}\overline{\text{CHD}}}> 1$, the SNP is proportionally more frequent in CHD than in non-CHD patients, hence the study favours a positive correlation between the presence of the SNP and the occurrence of CHD; if ${f}_{\text{SNPinCHD}}/{f}_{\text{SNPin}\overline{\text{CHD}}}\lt 1$, the SNP is proportionally less frequent in CHD than in non-CHD patients, hence the study favours a negative correlation; if ${f}_{\text{SNPinCHD}}/{f}_{\text{SNPin}\overline{\text{CHD}}}=1$, the SNP is equally frequent in CHD and in non-CHD patients, hence the study favours no correlation.

We plot this ratio of fractions for each study, grouped by ethnicity and CHD phenotype, in Fig. 4. We also plot the ratio for the combined data sets included in each panel. We observe that the ratio of the data sets are asymmetrical distributed about the ratio equal to one, showing a predominance of ratios smaller than one. The ratio of the combined data sets included in each panel is slightly smaller than one for the Caucasian studies (for both CHD phenotypes) and larger than one for the Asian studies. This asymmetry indicates heterogeneity in the studies, as also observed in the meta-analysis of Zhang et al. (2011).

In Fig. 5A, we plot this ratio of fractions as a function of the sample size. We observe that smaller data sets are distributed across a wide range of values of this ratio, whereas larger data sets are distributed more closely to one. This implies that smaller data sets favour either positive or negative correlation, whereas larger data sets favour no correlation.

### Hypotheses testing

First we test the hypothesis *H*_1_ that the presence of TNF-*α* SNP is related to the occurrence of CHD against the null hypothesis *H*_0_ that the presence of the SNP is unrelated to the occurrence of CHD. By the Bayes theorem, the probability of a hypothesis *H_i_* given the data *D*_SNP_ is the posterior probability of the corresponding hypothesis

(1) \begin{eqnarray*} \displaystyle P({H}_{i}\vert {D}_{\text{SNP}})=\frac{P({D}_{\text{SNP}}\vert {H}_{i})P({H}_{i})}{P({D}_{\text{SNP}})},&&\displaystyle \end{eqnarray*}

where *P*(*D*_SNP_|*H_i_*) is the evidence, *P*(*H_i_*) is the prior probability of *H_i_* and $P({D}_{\text{SNP}})=\sum _{n}P({D}_{\text{SNP}}\vert {H}_{i})P({H}_{i}).$ The subscript in *D*_SNP_ reminds us that the random variable is the occurrence of the SNP. In order to infer which hypothesis is more likely in view of the data, we compare the evidence computed for the two hypotheses. The evidence is the integral of the likelihood over the *j*-dimensional parameter space *p*_*i*,*j*_ of the hypothesis *H_i_*

(2) \begin{eqnarray*} \displaystyle P({D}_{\text{SNP}}\vert {H}_{i})=\int \nolimits {d}^{j}{p}_{i,j}~P({D}_{\text{SNP}}\vert {p}_{i,j},{H}_{i})P({p}_{k,n}\vert {H}_{i}).&&\displaystyle \end{eqnarray*}

Assuming equal prior probabilities for the two hypotheses, then from Eq. (1) it follows that

(3) \begin{eqnarray*} \displaystyle \frac{P({H}_{1}\vert {D}_{\text{SNP}})}{P({H}_{0}\vert {D}_{\text{SNP}})}=\frac{P({D}_{\text{SNP}}\vert {H}_{1})}{P({D}_{\text{SNP}}\vert {H}_{0})}.&&\displaystyle \end{eqnarray*}

We compute the evidence of the two hypotheses, for each data set separately and for the combined data sets grouped by CHD phenotype. We follow the procedure detailed in Vourvouhaki & Carvalho (2011), which we here summarize for one data set and then generalize for the combined data sets. In all cases, we choose a uniform distribution for the prior of the parameters, which is justified by the absence of an *a priori* bias on the values of the parameters (MacKay, 2003).

The evidence of *H*_0_, *P*(*D*_SNP_|*H*_0_), is computed assuming that the presence of the SNP is described by a binomial distribution with one parameter only, namely the probability *p*_0_ that the SNP occurs in a given population. For *n*_SNP_ occurrences of the SNP and ${n}_{\overline{\text{SNP}}}$ non-occurrences of the SNP in a sample of size $n={n}_{\text{SNP}}+{n}_{\overline{\text{SNP}}}$, the likelihood *P*(*D*_SNP_|*p*_0_, *H*_0_) is given by

(4) \begin{eqnarray*} \displaystyle P({D}_{\text{SNP}}\vert {p}_{0},{H}_{0})={p}_{0}^{{n}_{\text{SNP}}}(1-{p}_{0})^{{n}_{\overline{\text{SNP}}}}.&&\displaystyle \end{eqnarray*}

Moreover, assuming a uniform prior distribution for *p*_0_, *P*(*p*_0_) = 1, we find that

(5) \begin{eqnarray*} \displaystyle P({D}_{\text{SNP}}\vert {H}_{0})=\int \nolimits \nolimits _{0}^{1}d{p}_{0}~P({D}_{\text{SNP}}\vert {p}_{0},{H}_{0})P({p}_{0}\vert {H}_{0})=\frac{{n}_{\text{SNP}}!~{n}_{\overline{\text{SNP}}}!}{({n}_{\text{SNP}}+{n}_{\overline{\text{SNP}}}+1)!},&&\displaystyle \end{eqnarray*}

where *n*! stands for the factorial of *n*.

The evidence of *H*_1_, *P*(*D*_SNP_|*H*_1_), is computed assuming that the presence of the SNP is described by a binomial distribution with two parameters, namely the probability *p*_1,CHD_ that the SNP occurs in the subset of CHD patients and the probability ${p}_{1,\overline{\text{CHD}}}$ that the SNP occurs in the subset of non-CHD patients,

(6) \begin{eqnarray*} \displaystyle P({D}_{\text{SNP}}\vert {H}_{1})&=&\displaystyle \int \nolimits \nolimits _{0}^{1}d{p}_{1,\mathrm{CHD}}\int \nolimits \nolimits _{0}^{1}d{p}_{1,\overline{\text{CHD}}}\nonumber\\ \displaystyle &&\displaystyle \times \, P({D}_{\text{SNP}}\vert {p}_{1,\mathrm{CHD}},{p}_{1,\overline{\text{CHD}}},{H}_{1})P({p}_{1,\mathrm{CHD}},{p}_{1,\overline{\text{CHD}}}\vert {H}_{1}). \end{eqnarray*}

For *n*_SNP,CHD_ occurrences of the SNP and ${n}_{\overline{\text{SNP}},\text{CHD}}$ non-occurrences of the SNP in a subset of CHD patients ${n}_{\text{CHD}}={n}_{\text{SNP,CHD}}+{n}_{\overline{\text{SNP}},\text{CHD}}$, and also for ${n}_{\text{SNP},\overline{\text{CHD}}}$ occurrences of the SNP and ${n}_{\overline{\text{SNP}},\overline{\text{CHD}}}$ non-occurrences of the SNP in a subset of non-CHD patients ${n}_{\overline{\text{CHD}}}={n}_{\mathrm{SNP},\overline{\text{CHD}}}+{n}_{\overline{\text{SNP}},\overline{\text{CHD}}},$ the likelihood $P({D}_{\text{SNP}}\vert {p}_{1,\mathrm{CHD}},{p}_{1,\overline{\text{CHD}}},{H}_{1})$ is separable, i.e., it can be decomposed into the product of the likelihoods *P*(*D*_SNP_|*p*_1,CHD_, *H*_1_) and $P({D}_{\text{SNP}}\vert {p}_{1,\overline{\text{CHD}}},{H}_{1})$, as follows

(7) \begin{eqnarray*} \displaystyle P({D}_{\text{SNP}}\vert {p}_{1,\text{CHD}},{p}_{1,\overline{\text{CHD}}},{H}_{1})&=&\displaystyle {p}_{1,\text{CHD}}^{{n}_{\text{SNP, CHD}}}(1-{p}_{1,\text{CHD}})^{{n}_{\overline{\text{SNP}},\text{CHD}}}\nonumber\\ \displaystyle &&\displaystyle \times \, {p}_{1,\overline{\text{CHD}}}^{{n}_{\text{SNP},\overline{\text{CHD}}}}(1-{p}_{1,\overline{\text{CHD}}})^{{n}_{\overline{\text{SNP}},\overline{\text{CHD}}}}\nonumber\\ \displaystyle &\equiv &\displaystyle P({D}_{\text{SNP}}\vert {p}_{1,\text{CHD}},{H}_{1})P({D}_{\text{SNP}}\vert {p}_{1,\overline{\text{CHD}}},{H}_{1}). \end{eqnarray*}

Assuming a uniform probability for *p*_1,CHD_ and ${p}_{1,\overline{\text{CHD}}},$ $P({p}_{1,\text{CHD}},{p}_{1,\overline{\text{CHD}}}\vert {H}_{1})=$1 and moreover that the priors on *p*_1,CHD_ and ${p}_{1,\overline{\text{CHD}}}$ are separable, the posterior distribution will also be separable and given by

(8) \begin{eqnarray*} \displaystyle P({D}_{\text{SNP}}\vert {H}_{1})&=&\displaystyle \int \nolimits \nolimits _{0}^{1}d{p}_{1,\text{CHD}}~P({D}_{\text{SNP}}\vert {p}_{1,\text{CHD}},{H}_{1})P({p}_{1,\text{CHD}}\vert {H}_{1})\nonumber\\ \displaystyle &&\displaystyle \times \, \int \nolimits \nolimits _{0}^{1}d{p}_{1,\overline{\text{CHD}}}~P({D}_{\text{SNP}}\vert {p}_{1,\overline{\text{CHD}}},{H}_{1})P({p}_{1,\overline{\text{CHD}}}\vert {H}_{1})\nonumber\\ \displaystyle &=&\displaystyle \frac{{n}_{\text{SNP,CHD}}!~{n}_{\overline{\text{SNP}},\text{CHD}}!}{({n}_{\text{SNP,CHD}}+{n}_{\overline{\text{SNP}},\text{CHD}}+1)!}\frac{{n}_{\text{SNP},\overline{\text{CHD}}}!~{n}_{\overline{\text{SNP}},\overline{\text{CHD}}}!}{({n}_{\text{SNP},\overline{\text{CHD}}}+{n}_{\overline{\text{SNP}},\overline{\text{CHD}}}+1)!}. \end{eqnarray*}

In order to compare the hypotheses, we take the ratio of the corresponding evidences, *B*_10_ = *P*(*H*_1_|*D*)/*P*(*H*_0_|*D*), which we present in Table 1 (columns 7–8). This quantity is known as the Bayes factor and gives empirical levels of significance for the strength of the evidence of the test hypothesis over that of the null hypothesis. It also encapsulates the Occam’s factor, which measures the adequacy of a hypothesis to the data over the parameter space of the hypothesis (MacKay, 2003). The levels of significance ascribed to the Bayes factor are calibrated by the Jeffrey’s scale (Kass & Raftery, 1995). According to this scale, a Bayes factor larger than one indicates that *H*_1_ is favoured over *H*_0_. Otherwise, *H*_0_ is favoured over *H*_1_. For the data sets taken separately, the results from this hypothesis test mostly agree with the corresponding results presented in the meta-analysis by Chu et al. (2013, see Fig. 1).

**Figure 6:**
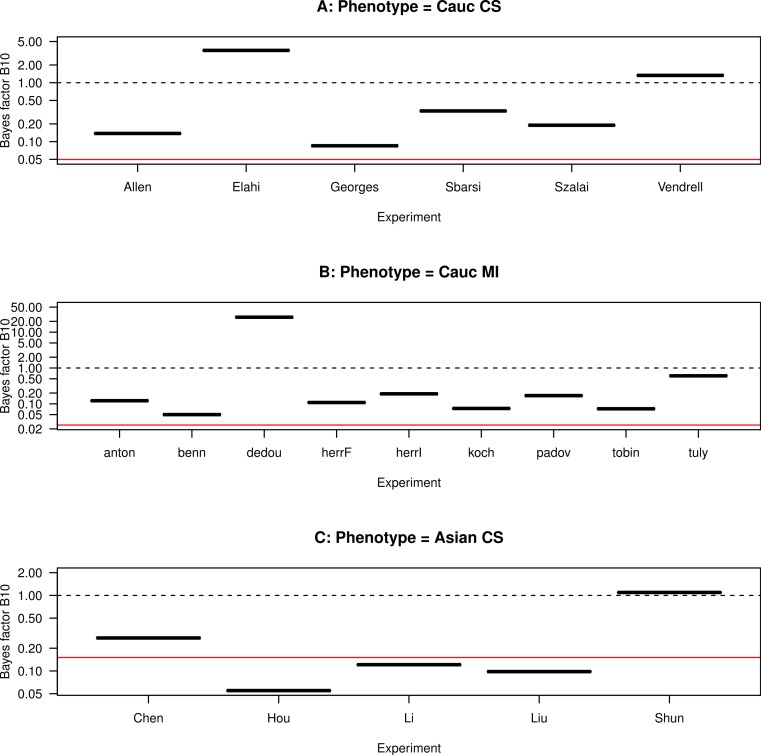
Funnel plot for the Bayes factor. The Bayes factor for each study, grouped by ethnicity and CHD phenotype. (A) Caucasians with coronary stenosis; (B) Caucasians with myocardial infarction; (C) Asians with coronary stenosis. The solid horizontal line marks the average Bayes factor of the data sets included in each panel. The dashed horizontal line marks the Bayes factor equal to one.

We plot the Bayes factor for each study, grouped by ethnicity and CHD phenotype, in Fig. 6. For the data sets taken separately, we observe that the Bayes factor is asymmetrically distributed about the Bayes factor equal to one, with most Bayes factors being smaller than one. The exceptions are Elahi et al. (2008), Vendrell et al. (2003) and Dedoussis et al. (2005) for the Caucasian population, and Shun et al. (2009) for the Asian population. This asymmetry indicates heterogeneity in the results. For the combined data sets included in each panel, the Bayes factor takes values 0.03–0.05 for the Caucasian population and 0.15 for the Asian population, which indicates that there is no evidence for *H*_1_ over *H*_0_. We also observe that, for the Caucasian population, the Bayes factor of the combined data sets is outside the range of variability of the Bayes factor of the data sets considered separately. This suggests that the combination of the Caucasian data sets causes a new data pattern to emerge. Conversely the combination of the Asian data sets leads to an approximately average data pattern. Hence we conclude that the data favour *H*_0_ over *H*_1_. Since *H*_0_ yields trivial results, in the subsequent subsections we present the results also for *H*_1_ to illustrate the application of the formalism to a more general setup. It is also instructive to compare the subsequent results using both hypotheses.

In Fig. 5B, we plot the Bayes factor as a function of the sample size. We observe that smaller data sets are distributed across a wide range of values of the Bayes factor, whereas larger data sets are distributed across values smaller than one. This implies that smaller data sets favour either *H*_0_ or *H*_1_, whereas larger data sets favour *H*_0_.

**Figure 7:**
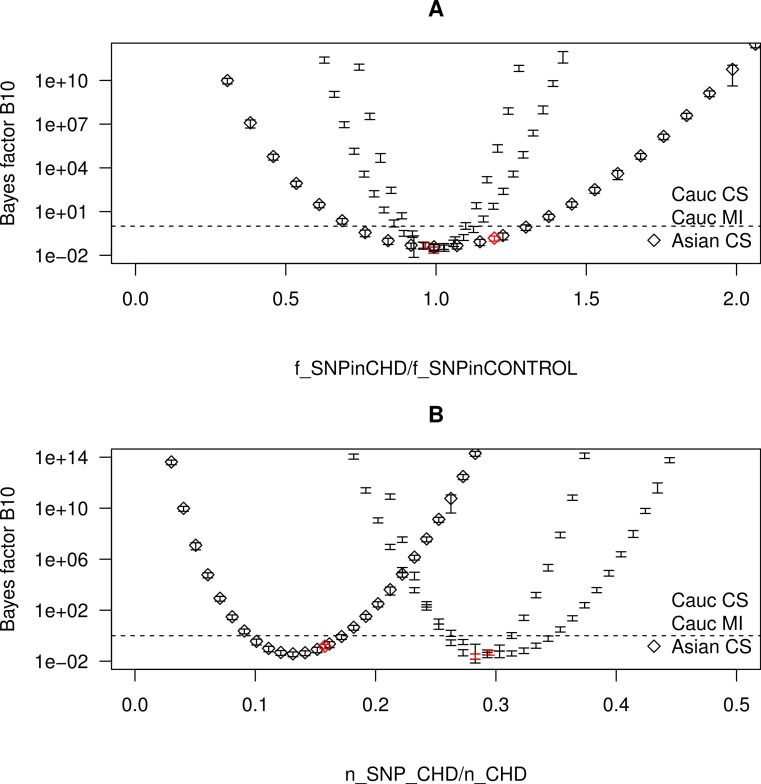
Bayes factor as a function of the frequency of SNP in the CHD populations. The Bayes factor for several realizations of CHD populations with the same *n*_CHD_ but with different fractions of SNP, grouped by ethnicity and CHD phenotype. The realizations that correspond to a real combined data set are marked as red points. The dashed horizontal line marks the Bayes factor equal to one. (A) The Bayes factor as a function of ${f}_{\text{SNPinCHD}}/{f}_{\text{SNPin}\overline{\text{CHD}}}$. (B) The Bayes factor as a function of *f*_SNPinCHD_.

#### Correlation sign

Comparing Fig. 6 with Fig. 4, we observe that, among the studies with Bayes factor larger than one, Elahi et al. (2008) has a ratio ${f}_{\text{SNPinCHD}}/{f}_{\text{SNPin}\overline{\text{CHD}}}\lt 1$, i.e., the SNP is proportionally less frequent in CHD than in non-CHD patients, which indicates a negative correlation between the presence SNP and the occurrence of CHD. Another example of comparatively large Bayes factor and low ratio ${f}_{\text{SNPinCHD}}/{f}_{\text{SNPin}\overline{\text{CHD}}}$ is the study of Tulyakova et al. (2004). This indicates that the hypotheses as formulated do not distinguish the correlation sign.

To further explore how the ratio ${f}_{\text{SNPinCHD}}/{f}_{\text{SNPin}\overline{\text{CHD}}}$ affects the result of the hypothesis testing, we consider several realizations of CHD populations with the same *n*_CHD_ but with different fractions of SNP. More specifically for each combined data set, we vary *n*_SNP,CHD_ while varying simultaneously ${n}_{\overline{\text{SNP}},\text{CHD}}$ so as to keep *n*_CHD_ constant. Throughout the different realizations, the size of the control population is kept equal to the size of the control population of the combined data sets grouped by ethnicity and CHD phenotype. For each realization, we compute both *f*_SNPinCHD_ (note that ${f}_{\text{SNPin}\overline{\text{CHD}}}$ is by construction kept fixed) and *B*_10_, and plot the results in Fig. 7. The realizations with the *f*_SNPinCHD_ of a real combined data set are marked as red points. In Fig. 7A, we plot *B*_10_ as a function of ${f}_{\text{SNPinCHD}}/{f}_{\text{SNPin}\overline{\text{CHD}}}$, from which there result three parabolae centred at the same point. In Fig. 7B, for a better visualization of the behaviour of *B*_10_, we plot *B*_10_ as a function of *f*_SNPinCHD_, from which there result three parabolae centred at different points. We observe that *B*_10_ follows a parabola, taking the minimum value when ${f}_{\text{SNPinCHD}}/{f}_{\text{SNPin}\overline{\text{CHD}}}=1$ and increasing in both directions with the increase of $\vert {f}_{\text{SNPinCHD}}/{f}_{\text{SNPin}\overline{\text{CHD}}}-1\vert $, i.e., with the increase of the distance from 1. This confirms that the hypotheses as formulated do not distinguish between a positive correlation of the SNP with CHD (${f}_{\text{SNPinCHD}}/{f}_{\text{SNPin}\overline{\text{CHD}}}> 1$) and a negative correlation (${f}_{\text{SNPinCHD}}/{f}_{\text{SNPin}\overline{\text{CHD}}}\lt 1$). Hence, the value of ${f}_{\text{SNPinCHD}}/{f}_{\text{SNPin}\overline{\text{CHD}}}$ complements the value of *B*_10_ in the characterization of the correlation.

## Results

### Inference of conditional probabilities

#### Posterior probability for the occurrence of CHD

We proceed to compute the probability for the occurrence of CHD, i.e., given the data on the presence of the SNP, we determine the probability that a patient has CHD. This is defined as the posterior probability

(9) \begin{eqnarray*} \displaystyle P(\text{CHD}\vert {D}_{\text{SNP}},{H}_{i})=\frac{P({D}_{\text{SNP}}\vert (\text{CHD,}{H}_{i})P(\text{CHD})}{P({D}_{\text{SNP}}\vert {H}_{i})}.&&\displaystyle \end{eqnarray*}

The prior probability *P*(CHD) is based on the available information on the occurrence of CHD. This probability can be computed by combining all the risk factors per age interval per pathology. According to the European guidelines, less than 4 in 1,000 people have CS (Hamm et al., 2011), whereas about 1 in 1,000 people have MI (Steg et al., 2012). We then use *P*(CHD) = 0.004 for CS and *P*(CHD) = 0.001 for MI.

The evidence *P*(*D*_SNP_|*H_i_*) can be decomposed as

(10) \begin{eqnarray*} \displaystyle P({D}_{\text{SNP}}\vert {H}_{i})=P({D}_{\text{SNP}}\vert (\text{CHD,}{H}_{i})P(\text{CHD})+P({D}_{\text{SNP}}\vert \overline{\text{CHD}},{H}_{i})P(\overline{\text{CHD}}).&&\displaystyle \end{eqnarray*}

In the case of *H*_0_,

\begin{eqnarray*} \displaystyle P({D}_{\text{SNP}}\vert (\text{CHD,}{H}_{0})=\left({n\atop {n}_{\text{SNP}}}\right){p}_{0}^{{n}_{\text{SNP}}}(1-{p}_{0})^{{n}_{\overline{\text{SNP}}}}\equiv P({D}_{\text{SNP}}\vert {H}_{0})&&\displaystyle \end{eqnarray*}

(11) \begin{eqnarray*} \displaystyle P({D}_{\text{SNP}}\vert \overline{\text{CHD}},{H}_{0})=P({D}_{\text{SNP}}\vert {H}_{0}),&&\displaystyle \end{eqnarray*}

whereas in the case of *H*_1_,

\begin{eqnarray*} \displaystyle P({D}_{\text{SNP}}\vert (\text{CHD,}{H}_{1})=\left({{n}_{\text{CHD}}\atop {n}_{\text{SNP,CHD}}}\right){p}_{1,\text{CHD}}^{{n}_{\text{SNP,CHD}}}(1-{p}_{1,C H D})^{{n}_{\overline{\text{SNP}},\text{CHD}}}&&\displaystyle \end{eqnarray*}

(12) \begin{eqnarray*} \displaystyle P({D}_{\text{SNP}}\vert \overline{\text{CHD}},{H}_{1})=\left({{n}_{\overline{\text{CHD}}}\atop {n}_{S N P,\overline{\text{CHD}}}}\right){p}_{1,\overline{\text{CHD}}}^{{n}_{\text{SNP},\overline{\text{CHD}}}}(1-{p}_{1,\overline{\text{CHD}}})^{{n}_{\overline{\text{SNP}},\overline{\text{CHD}}}}.&&\displaystyle \end{eqnarray*}

In the previous section, we computed the evidence by marginalizing the parameters of each hypothesis. Here, assuming a hypothesis *H_i_* and using the Bayes theorem, we compute the posterior probability of each parameter *p*_*i*,*j*_ given the data

(13) \begin{eqnarray*} \displaystyle P({p}_{i,j}\vert {D}_{\text{SNP}})=\frac{P({D}_{\text{SNP}}\vert {p}_{i,j})P({p}_{i,j}\vert {H}_{i})}{P({D}_{\text{SNP}}\vert {H}_{i})},&&\displaystyle \end{eqnarray*}

and find for *p*_*i*,*j*_ the value that maximizes the likelihood *P*(*D*_SNP_|*p*_*i*,*j*_). In the case of *H*_0_, we compute the posterior probability of the single parameter *p*_*i*,*j*_ = *p*_0_, where *P*(*D*_SNP_|*p*_0_) is given by Eq. (4), *P*(*D*_SNP_|*H*_0_) is given by Eq. (5) and *P*(*p*_0_|*H*_0_) is assumed uniform. Taking the derivative with respect to *p*_0_ and solving for *dP*(*p*_0_|*D*_SNP_)/*dp*_0_ = 0, we find for the maximum-likelihood value of *p*_0_ the value

(14) \begin{eqnarray*} \displaystyle {p}_{0(\text{maxL})}=\frac{{n}_{\text{SNP}}}{({n}_{\text{SNP}}+{n}_{\overline{\text{SNP}}})}.&&\displaystyle \end{eqnarray*}

Similarly in the case of *H*_1_, we compute the posterior probability of each of the two parameters ${p}_{i,j}=\{ {p}_{1,\text{CHD}},{p}_{1,\overline{\text{CHD}}}\} $, where *P*(*D*_SNP_|*p*_1,CHD_) and $P({D}_{\text{SNP}}\vert {p}_{1,\overline{\text{CHD}}})$ are given by Eq. (6), *P*(*D*_SNP_|*H*_1_) is given by Eq. (8) and both *P*(*p*_1,CHD_|*H*_1_) and $P({p}_{1,\overline{\text{CHD}}}\vert {H}_{1})$ are assumed uniform, finding for the maximum-likelihood values of *p*_1,CHD_ and ${p}_{1,\overline{\text{CHD}}}$ respectively

(15) \begin{eqnarray*} \displaystyle {p}_{1,\text{CHD}(\text{maxL})}=\frac{{n}_{\text{SNP,CHD}}}{({n}_{\text{SNP,CHD}}+{n}_{\overline{\text{SNP}},\text{CHD}})},&&\displaystyle \end{eqnarray*}

(16) \begin{eqnarray*} \displaystyle {p}_{1,\overline{\text{CHD}}(\text{maxL})}=\frac{{n}_{\text{SNP},\overline{\text{CHD}}}}{({n}_{\text{SNP},\overline{\text{CHD}}}+{n}_{\overline{\text{SNP}},\overline{\text{CHD}}})}.&&\displaystyle \end{eqnarray*}

Analogously we define the posterior probability

(17) \begin{eqnarray*} \displaystyle P(\overline{\text{CHD}}\vert {D}_{\text{SNP}},{H}_{i})=\frac{P({D}_{\text{SNP}}\vert \overline{\text{CHD}},{H}_{i})P(\overline{\text{CHD}})}{P({D}_{\text{SNP}}\vert {H}_{i})}.&&\displaystyle \end{eqnarray*}

Finally, using the maximum-likelihood value of *p*_*i*,*j*_, we compute *P*(CHD|*D*_SNP_, *H_i_*) for the data sets combined, which we present in Table 2.

In the case of *H*_0_, no information is added to the posterior probability, since by Eq. (11) the posterior probabilities equal the prior. Conversely in the case of *H*_1_, information is added to the posterior probability, since by Eq. (12) there result posterior probabilities different from the prior albeit compatible with the prior.

**Table 2:** Probabilities inferred from the combined data sets. To each hypothesis there correspond several rows consisting of (A) the parameters *p*_*i*,*j*_ given by the maximum-likelihood values, in particular, *p*_0_ (hence one row) in the case of *H*_0_, *p*_1,CHD_ and ${p}_{1,\overline{\text{CHD}}}$ (hence two rows) in the case of *H*_1_; (B) the posterior probability for the occurrence of (CHD, *P*(CHD|*D*_SNP_, *H_i_*) (hence one row for each hypothesis); (C) the predicted probabilities for the presence of the SNP, namely *P*(nextSNP, CHD|*D*_SNP_, *H_i_*), $P(\text{nextSNP},\overline{\text{CHD}}\vert {D}_{\text{SNP}},{H}_{i})$ and *P*(nextSNP|*D*_SNP_, *H_i_*) (hence three rows for each hypothesis); and (D) the probability ratio that measures the influence of CHD in the presence of the SNP, *r*_nextSNP,CHD_ ≡ *P*(nextSNP, CHD|*D*_SNP_, *H_i_*)/*P*(nextSNP|*D*_SNP_, *H_i_*), computed from the combined data of each phenotype (hence one row for each hypothesis). Column 1: The hypotheses. Column 2: The inferred quantities, as described above. Columns 3–5: The values of the inferred quantities for the combined ethnicity and CHD phenotype.

Hypothesis	Probabilities	Phenotype (*j*)		
		Cauc CS	Cauc MI	Asian CS
*H*_0_	*p*_0_	0.299 ± 0.001	0.284 ± 0.001	0.141 ± 0.001
	*P*(CHD|*D*_SNP_, *H*_0_)	(4.00 ± 1.31) ⋅ 10^−3^	(1.00 ± 0.25) ⋅ 10^−3^	(4.00 ± 0.91) ⋅ 10^−3^
	*P*(nextSNP, *CHD*|*D*_SNP_, *H*_0_)	(1.19 ± 0.39) ⋅ 10^−3^	(0.28 ± 0.07) ⋅ 10^−3^	(0.56 ± 0.13) ⋅ 10^−3^
	$P(\text{nextSNP},\overline{\text{CHD}}\vert {D}_{\text{SNP}},{H}_{0})$	0.298 ± 1.093	0.284 ± 1.752	0.141 ± 0.360
	*P*(nextSNP|*D*_SNP_, *H*_0_)	0.299 ± 1.093	0.284 ± 1.572	0.141 ± 0.360
	*r*_nextSNP,CHD_	(4.00 ± 14.65) ⋅ 10^−3^	(1.00 ± 5.54) ⋅ 10^−3^	(4.00 ± 10.22) ⋅ 10^−3^
*H*_1_	*p*_1,CHD_	0.295 ± 0.001	0.283 ± 0.001	0.158 ± 0.001
	${p}_{1,\overline{\text{CHD}}}$	0.305 ± 0.001	0.285 ± 0.001	0.132 ± 0.001
	*P*(CHD|*D*_SNP_, *H*_1_)	(3.42 ± 7.94) ⋅ 10^−3^	(0.98 ± 3.26) ⋅ 10^−3^	(5.00 ± 7.02) ⋅ 10^−3^
	*P*(nextSNP, CHD|*D*_SNP_, *H*_1_)	(1.00 ± 2.34) ⋅ 10^−3^	(0.28 ± 0.92) ⋅ 10^−3^	(0.79 ± 1.11) ⋅ 10^−3^
	$P(\text{nextSNP},\overline{\text{CHD}}\vert {D}_{\text{SNP}},{H}_{1})$	0.304 ± 0.598	0.285 ± 0.926	0.131 ± 0.244
	*P*(nextSNP|*D*_SNP_, *H*_1_)	0.305 ± 0.598	0.285 ± 0.926	0.132 ± 0.244
	*r*_nextSNP,CHD_	(3.30 ± 10.02) ⋅ 10^−3^	(0.98 ± 4.54) ⋅ 10^−3^	(6.00 ± 13.84) ⋅ 10^−3^

#### Prediction of the presence of the SNP

We now proceed to compute the probability for the presence of the SNP, i.e., given the data, we determine the probability that a randomly selected patient (with or without CHD) has the SNP. This probability is defined as

(18) \begin{eqnarray*} \displaystyle P(\text{nextSNP}\vert {D}_{\text{SNP}},{H}_{i})&=&\displaystyle P(\text{nextSNP}\vert {D}_{\text{SNP}},\text{CHD})P(\text{CHD}\vert {D}_{\text{SNP}},{H}_{i})\nonumber\\ \displaystyle &&\displaystyle +\, P(\text{nextSNP}\vert {D}_{\text{SNP}},\overline{\text{CHD}})P(\overline{\text{CHD}}\vert {D}_{\text{SNP}},{H}_{i})\nonumber\\ \displaystyle &\equiv &\displaystyle P(\text{nextSNP},\text{CHD}\vert {D}_{\text{SNP}},{H}_{i})+P(\text{nextSNP},\overline{\text{CHD}}\vert {D}_{\text{SNP}},{H}_{i}). \end{eqnarray*}

In the case of *H*_0_,

(19) \begin{eqnarray*} \displaystyle P(\text{nextSNP}\vert {D}_{\text{SNP}},\text{CHD})=P(\text{nextSNP}\vert {D}_{\text{SNP}},\overline{\text{CHD}})={p}_{0},&&\displaystyle \end{eqnarray*}

whereas in the case of *H*_1_,

\begin{eqnarray*} \displaystyle P(\text{nextSNP}\vert {D}_{\text{SNP}},\text{CHD})={p}_{1,\text{CHD}},& \end{eqnarray*}

(20) \begin{eqnarray*} \displaystyle P(\text{nextSNP}\vert {D}_{\text{SNP}},\overline{\text{CHD}})={p}_{1,\overline{\text{CHD}}}.&&\displaystyle \end{eqnarray*}

Using the maximum-likelihood values of *p*_*i*,*j*_ and the posterior probability *P*(CHD|*D*_SNP_, *H_i_*) computed above, we compute *P*(nextSNP|*D*_SNP_, *H_i_*), which we present in Table 2.

For completion, using the Bayes theorem, we invert *P*(nextSNP|*D*_SNP_, CHD) to find the probability that CHD will occur given that the SNP is present in a randomly selected patient

(21) \begin{eqnarray*} \displaystyle P(\text{CHD}\vert \text{nextSNP},{H}_{i})=\frac{P(\text{nextSNP}\vert {D}_{\text{SNP}},\text{CHD})P(\text{CHD}\vert {D}_{\text{SNP}},{H}_{i})}{P(\text{nextSNP}\vert {D}_{\text{SNP}},{H}_{i})}.&&\displaystyle \end{eqnarray*}

Similarly, inverting $P(\text{nextSNP}\vert {D}_{\text{SNP}},\overline{\text{CHD}})$, we find the probability that CHD will not occur given that the SNP is present in a randomly selected patient, $P(\overline{\text{CHD}}\vert \text{nextSNP},{H}_{i})$, which can be found simply by replacing CHD by $\overline{\text{CHD}}$ in Eq. (21).

In order to quantify the influence of CHD in the presence of the SNP, we compute the ratio of *P*(nextSNP, CHD|*D*_SNP_) to *P*(nextSNP|*D*_SNP_, *H_i_*), which gives an estimate of how much the occurrence of CHD indicates the presence of the SNP. This is also the probability in Eq. (21). In the case of *H*_0_, this ratio equals the posterior probability of occurrence of CHD. Conversely in the case of *H*_1_, this ratio is different from the posterior probability of occurrence of CHD albeit compatible with it. The occurrence of CHD indicates the presence of the SNP in of order 0.1% of patients (0.1–0.4% in the case of *H*_0_, 0.1–0.6% in the case of *H*_1_), which suggests that the occurrence of CHD is not a good marker for the presence of the SNP.

In order to quantify the influence of the SNP in the occurrence of (CHD, we compute the ratio of *P*(CHD|nextSNP, *H_i_*) to *P*(CHD|*D*_SNP_, *H_i_*), which gives an estimate of how much the presence of the SNP indicates the occurrence of CHD. This is also the probability in Eqs. (19) and (20). The presence of SNP indicates the occurrence of CHD in of order 0.1% of patients (0.141–0.299% in the case of *H*_0_, 0.158–0.295% in the case of *H*_1_), which suggests that the presence of the SNP is not a risk factor for the emergence of CHD.

**Table 3:** Probabilities inferred from the combined data sets excluding the low-significance data sets and the data sets with extreme results. Excluded: Elahi et al. (2008), Dedoussis et al. (2005) and Chen et al. (2001). Similarly to Table 2, to each hypothesis there correspond several rows consisting of: (A) the parameters given by the maximum-likelihood values (one row in the case of *H*_0_ and two rows in the case of *H*_1_); (B) the posterior probability for the occurrence of CHD (one row for each hypothesis); (C) the predicted probabilities for the presence of the SNP (three rows for each hypothesis); and (D) the probability ratio that measures the influence of CHD in the presence of the SNP, computed from the combined data of each phenotype (one row for each hypothesis). Column 1: The hypotheses. Column 2: The inferred quantities, as described above. Columns 3–5: The values of the inferred quantities for the combined ethnicity and CHD phenotype.

Hypothesis	Probabilities	Phenotype (*j*)		
		Cauc CS	Cauc MI	Asian CS
*H*_0_	*p*_0_	0.288 ± 0.001	0.296 ± 0.001	0.136 ± 0.001
	*P*(CHD|*D*_SNP_, *H*_0_)	(4.00 ± 1.26) ⋅ 10^−3^	(1.00 ± 0.24) ⋅ 10^−3^	(4.00 ± 0.89) ⋅ 10^−3^
	*P*(nextSNP, CHD|*D*_SNP_, *H*_0_)	(1.15 ± 0.36) ⋅ 10^−3^	(0.30 ± 0.07) ⋅ 10^−3^	(0.55 ± 0.12) ⋅ 10^−3^
	$P(\text{nextSNP},\overline{\text{CHD}}\vert {D}_{\text{SNP}},{H}_{0})$	0.287 ± 1.018	0.296 ± 1.605	0.136 ± 0.340
	*P*(nextSNP|*D*_SNP_, *H*_0_)	0.289 ± 1.018	0.296 ± 1.605	0.136 ± 0.340
	*r*_nextSNP,CHD_	(4.00 ± 14.16) ⋅ 10^−3^	(1.00 ± 5.54) ⋅ 10^−3^	(4.00 ± 10.00) ⋅ 10^−3^
*H*_1_	*p*_1,CHD_	0.290 ± 0.001	0.292 ± 0.001	0.151 ± 0.001
	${p}_{1,\overline{\text{CHD}}}$	0.287 ± 0.001	0.300 ± 0.001	0.128 ± 0.001
		*P*(CHD|*D*_SNP_, *H*_1_)	(3.34 ± 7.57) ⋅ 10^−3^	(0.99 ± 3.18) ⋅ 10^−3^	(5.11 ± 6.96) ⋅ 10^−3^
	*P*(nextSNP, CHD|*D*_SNP_, *H*_1_)	(0.97 ± 2.19) ⋅ 10^−3^	(0.29 ± 0.93) ⋅ 10^−3^	(0.77 ± 1.05) ⋅ 10^−3^
	$P(\text{nextSNP},\overline{\text{CHD}}\vert {D}_{\text{SNP}},{H}_{1})$	0.286 ± 0.542	0.300 ± 0.947	0.128 ± 0.234
	*P*(nextSNP|*D*_SNP_, *H*_1_)	0.287 ± 0.543	0.300 ± 0.947	0.129 ± 0.234
	*r*_nextSNP,CHD_	(3.38 ± 9.96) ⋅ 10^−3^	(0.96 ± 4.34) ⋅ 10^−3^	(6.02 ± 13.67) ⋅ 10^−3^

**Table 4:** Probabilities inferred from the combined data sets excluding the extreme data sets. Excluded: Georges et al. (2003), Bennet et al. (2006) and Hou et al. (2009). Similarly to Table 2, to each hypothesis there correspond several rows consisting of: (A) the parameters given by the maximum-likelihood values (one row in the case of *H*_0_ and two rows in the case of *H*_1_); (B) the posterior probability for the occurrence of CHD (one row for each hypothesis); (C) the predicted probabilities for the presence of the SNP (three rows for each hypothesis); and (D) the probability ratio that measures the influence of CHD in the presence of the SNP, computed from the combined data of each phenotype (one row for each hypothesis). Column 1: The hypotheses. Column 2: The inferred quantities, as described above. Columns 3–5: The values of the inferred quantities for the combined ethnicity and CHD phenotype.

Hypothesis	Probabilities	Phenotype (*j*)		
		Cauc CS	Cauc MI	Asian CS
*H*_0_	*p*_0_	0.308 ± 0.001	0.271 ± 0.001	0.177 ± 0.001
	*P*(CHD|*D*_SNP_, *H*_0_)	(4.00 ± 1.08) ⋅ 10^−3^	(1.00 ± 0.20) ⋅ 10^−3^	(4.00 ± 0.63) ⋅ 10^−3^
	*P*(nextSNP, CHD|*D*_SNP_, *H*_0_)	(1.12 ± 0.33) ⋅ 10^−3^	(0.27 ± 0.05) ⋅ 10^−3^	(0.71 ± 0.11) ⋅ 10^−3^
	$P(\text{nextSNP},\overline{\text{CHD}}\vert {D}_{\text{SNP}},{H}_{0})$	0.306 ± 0.923	0.270 ± 1.220	0.177 ± 0.314
	*P*(nextSNP|*D*_SNP_, *H*_0_)	0.308 ± 0.923	0.271 ± 1.220	0.177 ± 0.314
	*r*_nextSNP,CHD_	(4.00 ± 12.05) ⋅ 10^−3^	(1.00 ± 4.51) ⋅ 10^−3^	(4.00 ± 7.11) ⋅ 10^−3^
*H*_1_	*p*_1,CHD_	0.306 ± 0.001	0.270 ± 0.001	0.190 ± 0.001
	${p}_{1,\overline{\text{CHD}}}$	0.309 ± 0.001	0.271 ± 0.001	0.165 ± 0.001
	*P*(CHD|*D*_SNP_, *H*_1_)	(3.93 ± 6.97) ⋅ 10^−3^	(0.92 ± 2.58) ⋅ 10^−3^	(3.90 ± 4.35) ⋅ 10^−3^
	*P*(nextSNP, CHD|*D*_SNP_, *H*_1_)	(1.20 ± 2.14) ⋅ 10^−3^	(0.25 ± 0.70) ⋅ 10^−3^	(0.74 ± 0.828) ⋅ 10^−3^
	$P(\text{nextSNP},\overline{\text{CHD}}\vert {D}_{\text{SNP}},{H}_{1})$	0.308 ± 0.535	0.271 ± 0.698	0.165 ± 0.187
	*P*(nextSNP|*D*_SNP_, *H*_1_)	0.309 ± 0.535	0.271 ± 0.698	0.165 ± 0.187
	*r*_nextSNP,CHD_	(3.90 ± 9.68) ⋅ 10^−3^	(0.91 ± 3.48) ⋅ 10^−3^	(4.48 ± 7.13) ⋅ 10^−3^

### Sensitivity of the results

To test the robustness of this meta-analysis, we conceive two tests of the sensitivity of the results, namely to low-significance data sets, to data sets with extreme results and to extreme data sets.

To test the sensitivity of the results to low-significance data sets, we exclude the data sets with comparatively small sample sizes for the same CHD phenotype, namely the study by Elahi et al. (2008) and the study by Chen et al. (2001), from the combination. We also exclude the studies with extreme results (i.e., the studies with the largest Bayes factor), namely the study in Dedoussis et al. (2005). We recompute both the Bayes factors (Table 1) and the probabilities of CHD (Table 3). We observe that the Bayes factor in the new combination changes by 18%, −38% and 24%, respectively for the CS Caucasian, the MI Caucasian and the CS Asian population. The inferred parameters and probabilities vary by −6 to 6%, −5 to 2%, and −1 to 4%, respectively for the CS Caucasian, the MI Caucasian and the CS Asian population. The largest difference is observed for the CS Caucasian population due to the exclusion of the study by Elahi et al. (2008). The exclusion of the study by Dedoussis et al. (2005) from the MI Caucasian population causes predominantly negative differences.

To test the sensitivity of the results to extreme data sets, we exclude the data sets with comparatively large samples sizes for the same CHD phenotype, namely the study by Georges et al. (2003), the study by Bennet et al. (2006) and the study by Hou et al. (2009), from the combination. These are also the studies with the smallest Bayes factor for each CHD phenotype. We recompute both the Bayes factors (Table 1) and the probabilities of CHD (Table 4). We observe that the Bayes factor in the new combination changes by 3%, −19% and 32%, respectively for the CS Caucasian, the MI Caucasian and the CS Asian population. The inferred parameters and probabilities vary by −20 to −1%, 5 to 11%, and −26 to 25%, respectively for the CS Caucasian, the MI Caucasian and the CS Asian population. The largest difference is observed for the CS Asian population due to the exclusion of the study by Hou et al. (2009). The exclusion of the study by Georges et al. (2003) from the CS Caucasian population causes predominantly negative differences.

In both tests, the differences in the Bayes factor leave the result of the hypothesis testings unchanged, while the differences in the inferred parameters and probabilities also leave the conclusions unchanged. We thus infer that this formalism is largely insensitive to (a) low-significante data sets combined with data with extreme results, and to (b) extreme data sets, which renders this formalism significantly robust.

## Conclusions

In this manuscript we investigated the correlation between the occurrence of CHD with the presence of the −308 TNF-*α* SNP from fifteen independent data sets on Caucasians for two CHD phenotypes and from five independent data sets on Asian for one CHD phenotype. We showed how to combine independent data sets and to infer correlations using Bayesian analysis.

Hypothesis testing on the combined data sets indicated that there is no evidence for a correlation between the occurrence of CHD and the presence of the SNP, either on Caucasians or on Asians. This result agrees with previous meta-analyses (Zhang et al., 2011; Chu et al., 2013). As a measure of an eventual correlation, we computed the conditional probability of CHD given the SNP, normalized to the probability that CHD occurs, finding that the presence of the SNP indicates the occurrence of CHD in of order 0.1% of patients, i.e., in of order 0.1% of the occurrence of CHD is concomitant with the presence of SNP. We also tested the sensitivity of the results by excluding selected data sets from the meta-analysis. We found changes of order 10%, leaving the results unchanged and thus establishing this formalism as significantly robust.

An interesting extension of this work for the sake of completion is the inclusion of studies referring to Africans and Indians which are currently too few to extract convincing results.
